# Effectiveness of Physiotherapy and Occupational Therapy after Traumatic Brain Injury in the Intensive Care Unit

**DOI:** 10.1155/2012/768456

**Published:** 2012-04-05

**Authors:** Stephanie Hellweg

**Affiliations:** Departement of Neurorehabilitation, Rehaklinik Bellikon, Mutschellenstrasse 2, 5454 Bellikon, Switzerland

## Abstract

Physiotherapy and occupational therapy are frequently administered in intensive care units (ICUs) after traumatic brain injury (TBI) to promote recovery. The increasing economic pressure and the growing need for evidence of therapeutic effectiveness are reasons for reviewing the currently available scientific data. The databases of OTseeker, PEDro, Medline, and Cochrane were searched for studies on frequently applied therapeutic procedures in the ICU following a TBI. It becomes evident that the currently available data on physiotherapy and occupational therapy are very limited. Consequently, it is not possible to give conclusive recommendations within an evidence-based context. Studies of other neurological disorders indicate that early mobilisation positively influences outcome parameters such as the ability to walk. It can be concluded from three studies that physiotherapy for the prevention or treatment of ventilator-associated pneumonia is not effective. The proof of effectiveness for other physiotherapeutic and occupational therapy interventions must still be demonstrated.

## 1. Introduction

In Switzerland, about 11000 individuals of working age covered by obligatory accident insurance sustain a traumatic brain injury every year. Of these, 5–8% of the injuries are severe TBIs [1] with long-lasting or permanent disability. The most common causes are traffic accidents, followed by sports and recreational accidents. Within this group, young working males constitute a large subgroup. Thus, rehabilitation and subsequent vocational reintegration of the affected individuals is of high socioeconomic significance. 

Both insurance companies and the patients' environments increasingly demand the integration of scientific evidence into the rehabilitative process. This paper provides an overview of the current physiotherapeutic and occupational therapeutic methods administered to TBI survivors in the ICU. However, there is a paucity of published evidence due to the fact that the group of patients with severe TBI is relatively small. Thus, many studies are done with mixed patient groups. A general overview of evidence-based physiotherapy following a TBI can be found in Hellweg and Johannes [2].

The consequences of TBI can be far-reaching and vary depending on the type and location of the injuries. In comparison to strokes, which result in local damage to the brain due to circulation disruptions, accidents often involve injuries that are diffuse to high acceleration forces. They are also frequently combined with other injuries to internal organs or the locomotor system. This leads to a high degree of heterogeneity in the manifestation of clinical symptoms and makes the treatment of the accident victims particularly challenging.

In contrast to stroke patients, who are treated in stroke units in a standardised and interdisciplinary concept [3], there is no nationwide network of TBI units in Germany, Austria, and Switzerland that would be responsible for the primary care, intensive care, and early rehabilitation of individuals with traumatic brain injuries. Individuals with TBI in Switzerland are mostly treated in the surgical ICUs of university hospitals and specific regional hospitals in a nonstandardised interdisciplinary way. In Germany the “Bundesarbeitsgemeinschaft für Rehabilitation” (BAR), a federal institution to define standards in rehabilitation, has published recommendations for early rehabilitation of neurological patients including TBI patients in 1995. These recommendations have influenced the OPS 8-552 in the German DRG-system. The German DRG-system defines a “minimum standard” to treat neurological/neurosurgical early rehabilitation patients (e.g., minimum of 300 min therapy/day consisting of physiotherapy, occupational therapy, etc.) [4]. However, while these standards define the duration of therapy, they do not specify its methods.

The currently practised physiotherapy and occupational therapy in ICUs for individuals with brain injuries are primarily focused on structure and body functions such as respiratory therapy, passive-assistive movement for contracture prophylaxis, stimulation therapy, low-dose strength, and endurance training and stretching. Some of the interventions affect activities such as mobilisation into the seated position, into the wheelchair, and/or into a standing position, as well as practising self-care or ADL training.

The therapeutic goal is frequently focused on the prevention of secondary damage such as pneumonia or contractures, the promotion of consciousness and sensory perception and strengthening of the muscles. The overall aim is to achieve the highest possible degree of mobility and independence with regard to self-care.

## 2. Materials and Methods

A literature search was performed between August and November 2011 using the electronic databases Occupational Therapy Systematic Evaluation of Effectiveness Database (OTseeker), Physiotherapy Evidence Database (PEDro), Medline, and Cochrane Review Center.

The following studies were included:

- RCT, reviews or meta-analyses,
- >50% of patients with TBI,
- acute phase and/or ICU.

The following studies were excluded:

- focus on children and adolescents,
- no physiotherapeutic or occupational therapy intervention,
- mild or moderate degree of brain injuries,
- postacute phase, intervention not started in the early phase.

The key search terms were as follows: in OTseeker, PEDro, and Cochrane Review Center: “brain injury” without further specification; in Medline: a combination of “traumatic brain injury” OR “head injury,” “ICU” OR “IMC,” “physical therapy” OR physiotherapy and occupational therapy.

Due to the limited availability of studies, the above-mentioned data bases were also specifically searched for the following topics:

1. sensory/basal stimulation,
2. prevention of secondary complications:
  - respiratory therapy,
  - contracture prophylaxis,
3. serial casting,
4. mobilisation/verticalisation,
5. ADL training,
6. therapy intensity.

In cases where no studies fulfilled the defined criteria, studies for other patient groups were included.

The definition of the term “early” presented a challenge for this work because it is defined very differently in the literature, ranging from the first 24 hours up to 6 months following the accident. In order to differentiate this paper from other existing papers in the area of treatment of individuals with TBI [2], this work focuses on the area of ICUs.

## 3. Results and Discussion

### 3.1. Sensory/Basal Stimulation

Sensory/basal stimulation refers to the application of specific structured stimuli such as tactile, proprioceptive, vestibular, auditory, visual, or olfactory stimuli. Sensory/basal stimulation programmes differ significantly from each other with respect to duration and in their mode of stimulation (unimodal versus multimodal).

Sensory/basal stimulation programmes exist for comatose patients or patients in states ranging from vegetative to minimally conscious. The goal of sensory/basal stimulation is the activation of the brain, an improved stimulus transmission, and overall a quicker and better recovery of the level of consciousness. Although stimulation programmes are widespread in rehabilitation, the efficacy of these programmes has not been proven indisputably [5, 6]. The diversity of terms in the area of reduced states of consciousness with their corresponding inconsistent use is a massive problem from a scientific point of view, which is why studies are not comparable or hardly comparable. Furthermore, the quality of studies in this area can be considered low for the most part.

A randomised controlled trial study by Abbasi et al. from Iran [7] is available for the acute phase, respectively, the treatment period in the ICU. It deals with the question whether contact between the patient and his/her family is capable of positively influencing the degree of consciousness. The control group obtained standard care which limited the contact to the use of monitors or windows. The study group received structured stimulation by the family. In comparison to the control group, the study group showed an increased degree of consciousness as a positive study result. It is notable that the significance of this study for Europe is very questionable because a contact of more than 15 minutes per day between patients and relatives is commonly supported. The data from Iran would speak in favour of a more structured integration of family members in the rehabilitation process.

In conclusion, sensory/basal stimulation has not been proven to be effective for the treatment of TBI survivors.

### 3.2. Prevention of Secondary Complications

#### 3.2.1. Respiratory Therapy

Respiratory therapy is an important component of physiotherapy in ICUs. The therapeutic interventions are versatile and focus on various goals such as the promotion of alveolar ventilation, secretolysis, improved oxygen saturation, maintenance, and/or improvement of thorax mobility and the improvement of resilience.

In a randomised controlled trial of individuals with acquired brain injury, Patman et al. [8] researched whether respiratory therapy had a positive effect on ventilator-associated pneumonia and whether it leads to a reduction in the length of mechanical respiration, a shorter length of stay in the ICU, or prevention of ventilator-associated pneumonia. Though this is a widespread intervention, its efficacy could not be confirmed.

Ntoumenopoulos et al. [9] and Templeton and Palazzo [10] come to a similar conclusion for critically ill patients.

Respiratory therapy should be critically evaluated for patients who are subject to mechanical respiration.

#### 3.2.2. Contracture Prophylaxis

A common complication following a TBI are contractures. Contractures can be defined as a loss of joint mobility due to structural changes of muscles, tendons, and ligaments and other nonbony structures. The performance of daily routine tasks can be significantly impaired due to the prevalence of contractures.

Although the passive movement of joints and stretching are standard treatments performed by physiotherapists for patients with TBI in the ICU, no randomised controlled trial could be found for this method. This is most likely due to the patients' rather brief length of stay in the ICU. In addition, mobility is seldom limited in the long term and contractures occur relatively infrequently.

Katalinic et al. [11] published a Cochrane Review in which they studied the impact of stretching (including prolonged stretching by means of casting) as a treatment and prevention intervention for contractures for both neurological patients and musculoskeletal patients. The authors came to the conclusion that stretching has no clinically significant effect on joint mobility and the quality of life. In addition, stretching has little or no effect with regard to secondary endpoints such as pain, spasticity, activity limitations, and participation.

### 3.3. Serial Casting

Serial casting is widely used to reduce spastic hypertonia and to improve the range of motion in neurological postacute rehabilitation. Usually serial casts are applied that need to be changed in an interval of 4 to 7 days with the primary goal of improving the mobility of a joint.

The lack of existing studies that investigate the efficacy of this intervention in the ICU is likely due to the brief length of stay in the ICU.

Results of studies in the postacute stage [6, 12–16] come to the conclusion that this therapy does not result in a verifiable functional gain. However, passive joint mobility can be improved by serial casting. A follow-up treatment with splints is often indispensable, which is impressively evidenced in a paper by Moseley et al., [15]: serial casting results showed an average improvement of elbow mobility by 22 degrees. However, this range of motion already decreased by 11 degrees on the subsequent day and completely disappeared after 4 weeks. Follow-up treatment with splints was not performed in this study. In the German-speaking region, a widespread practice is to apply splints following the use of serial casting, which might improve results [17]. A reduction of hypertonia through serial casting cannot be proven on the basis of current scientific data.

### 3.4. Mobilisation/Verticalisation

Bed rest and deep sedation are common in ICUs. A number of studies [18–20] indicate that immobilisation leads to various negative effects on a musculoskeletal, pulmonary, cardiovascular, and endocrine/metabolic level such as neuromuscular weakness, muscle atrophies, pressure sores, atelectases and pneumonia, orthostatic dysregulation, and disturbed microvascularisation.

The term “mobilisation” is used in very different ways. In this paper, mobilisation means bringing the patient into an upright seated position at the edge of the bed or outside the bed or to a standing position. There is a controversial discussion about how early patients should be mobilised from the bed and be verticalised.

There is no existing data on the point in time when mobilisation is sensible for individuals with traumatic brain injuries.

In stroke patients, a very early rehabilitation trial, the AVERT-Study [21, 22], shows that an early mobilisation within the first 24 hours after stroke reduces mortality and long-term disability. This approach is increasingly used in poststroke rehabilitation. In addition, Indredavik et al. [3, 23] was able to provide evidence that early mobilisation in stroke units leads to decreased mortality rate, shortened duration of stay in the hospital, and more frequent return to the home environment. Multiple studies confirm that early mobilisation improves the patient's ability to walk [22, 24]. Three studies were found, whose results highlight the safety and feasibility of early mobilisation within the context of ICUs.

An Australian survey by Chang et al. [25] investigated whether the tilt table is used by physiotherapists in the ICU. The result of the survey indicated that the tilt table is frequently used as a method of mobilisation, especially for patients with severe disabilities, in order to improve musculoskeletal functions and to promote consciousness.

Positive effects of early verticalisation in ICUs were demonstrated for various clinical symptoms. Schweickert et al. [26] conducted a noteworthy study in which he researched the effect of early physiotherapy and occupational therapy while interrupting the sedation in a randomised controlled trial with 104 patients who required mechanical respiration. The control group received therapy according to the orders of the primary care team. The experimental group benefitted from a better functional outcome when released from the ICU, shorter amount of time spent in delirium, and shorter periods of mechanical respiration.

Morris et al. [27] were able to prove in a prospective cohort study that initiating a mobility protocol within the first 48 hours following mechanical respiration for patients with acute respiratory failure decreased both the duration of stay in the ICU and the total stay in the hospital.

Especially the last two studies mentioned highlight the fact that early mobilisation for intensive care patients is both feasible and safe.

In light of these findings the duration of bed rest and sedation in the ICU should be critically appraised.

Luther et al. [28] compared the tolerance of conventional standing exercise with the standing exercise on the therapy device “Erigo” which integrates stepping movements of the legs during verticalisation. The study group was small (*n* = 9) and consisted of unconscious patients in Bad Aibling, Germany within the first three months following brain injury. The tolerance was primarily measured by the frequency of syncope/presyncope during standing. The results indicate that the patients on the Erigo exhibit significantly less syncopes during verticalisation.

### 3.5. ADL Training/Self-Care

Training of self-care activities and activities of daily living (ADL) is frequently administered to patients with sensory, motor, or cognitive dysfunction. Some ICUs already start with this intervention at a very early point in time.

Bowen et al. evaluated such nonpharmacological interventions for sensory dysfunction due to a stroke or an acquired brain injury in their 2011 Cochrane Review [29]. ADL improvements were measured as outcome parameter. The authors come to the conclusion that it is not possible to make a statement on the effectiveness of this measure on the basis of the currently available data. In summary, the evidence of the efficacy for ADL training in the ICU is still to be determined.

### 3.6. Therapy Intensity

To evaluate the effect of therapy intensity, standard therapy programmes are compared with therapy programmes that are more intensive, that is, more time consuming. The results of the studies [6, 30–34] are consistent: more intensive rehabilitation programmes lead to earlier functional abilities.

In concrete terms, this means that more therapy results in earlier functional improvements.

It is important to note that most studies exclude comatose patients. The general requirement for the patients who were included was that they already exhibited minimal reactions to their environment.

The results by Zhu et al. [33, 34], who exclusively included individuals with traumatic brain injuries in their study, are especially interesting. Hellweg come to the conclusion that early intensive rehabilitation significantly improves the functional outcome of the first months following the accident but that no difference can be observed at the end of the rehabilitation. No additional studies could be found for the very early phase within the context of ICUs.

## 4. Conclusion

Current scientific data for the effectiveness of physiotherapy and occupational therapy after TBI in the ICU is very limited. Consequently, it is not possible to offer clear evidence-based recommendations. This is at least partially due to the brief length of stay in the ICU, which makes it difficult to investigate long-term outcome parameters. Studies conducted on other neurological disorders indicate that early mobilisation positively influences outcome parameters such as the ability to walk in the long term. Respiratory physiotherapy has not been shown to be effective for the prevention or treatment of ventilator-associated pneumonia. The efficacy of other physiotherapeutic and occupational treatment interventions must still be demonstrated.
